# The cyanobacterial endosymbiont of the unicellular algae *Rhopalodia gibba *shows reductive genome evolution

**DOI:** 10.1186/1471-2148-8-30

**Published:** 2008-01-28

**Authors:** Christoph Kneip, Christine Voβ, Peter J Lockhart, Uwe G Maier

**Affiliations:** 1 Department of Cell Biology, Philipps-University Marburg, Marburg, Germany; 2 Present address: Department of Molecular Biology, Max-Planck-Institute for Infection Biology, Berlin, Germany; 3 Allan Wilson Centre for Molecular Ecology and Evolution, Institute of Molecular BioSciences, Massey University, Palmerston North, New Zealand

## Abstract

**Background:**

Bacteria occur in facultative association and intracellular symbiosis with a diversity of eukaryotic hosts. Recently, we have helped to characterise an intracellular nitrogen fixing bacterium, the so-called spheroid body, located within the diatom *Rhopalodia gibba*. Spheroid bodies are of cyanobacterial origin and exhibit features that suggest physiological adaptation to their intracellular life style. To investigate the genome modifications that have accompanied the process of endosymbiosis, here we compare gene structure, content and organisation in spheroid body and cyanobacterial genomes.

**Results:**

Comparison of the spheroid body's genome sequence with corresponding regions of near free-living relatives indicates that multiple modifications have occurred in the endosymbiont's genome. These include localised changes that have led to elimination of some genes. This gene loss has been accompanied either by deletion of the respective DNA region or replacement with non-coding DNA that is AT rich in composition. In addition, genome modifications have led to the fusion and truncation of genes. We also report that in the spheroid body's genome there is an accumulation of deleterious mutations in genes for cell wall biosynthesis and processes controlled by transposases. Interestingly, the formation of pseudogenes in the spheroid body has occurred in the presence of intact, and presumably functional, *rec*A and *rec*F genes. This is in contrast to the situation in most investigated obligate intracellular bacterium-eukaryote symbioses, where at least either *rec*A or *rec*F has been eliminated.

**Conclusion:**

Our analyses suggest highly specific targeting/loss of individual genes during the process of genome reduction and establishment of a cyanobacterial endosymbiont inside a eukaryotic cell. Our findings confirm, at the genome level, earlier speculation on the obligate intracellular status of the spheroid body in *Rhopalodia gibba*. This association is the first example of an obligate cyanobacterial symbiosis involving nitrogen fixation for which genomic data are available. It represents a new model system to study molecular adaptations of genome evolution that accompany a switch from free-living to intracellular existence.

## Background

A diversity of extracellular and intracellular symbiotic interactions occurs between bacteria and eukaryote hosts. The degree of interconnection between partners ranges from the weak dependence of some extracellular associations to permanent or obligate intracellular symbiosis. In the latter case, the endosymbiont is transmitted vertically to the next generation without any need for re-infection. The dependence on the host can be stabilised by loss or inactivation of genes, whose products are no longer required in the partnership [1,2]. Consequently, intracellular obligate symbionts loose their autonomy and therefore the capacity for a host-independent life style. Isolated from free-living populations, vertically transmitted endosymbionts have limited possibilities for genetic exchange through processes such as conjugation or transformation. Typically, endosymbiont genes diverge rapidly in comparison to their homologues in free-living relatives, a phenomenon that perhaps reflects genetic drift operating on small population size [3,4] and/or relaxation of structural/functional constraints on endosymbiont protein evolution [5]. The genomes of obligate intracellular bacteria often show an accumulation of deleterious mutations and a higher AT-ratio, accompanied with reduction in genome size when compared to their free-living relatives [6]. The dimension of these processes can be as extreme as seen in the reduced genome of *Buchnera *sp., an endosymbiont of aphids with a genome size of 641 kbp [7,8] and *Carsonella*, a γ-proteobacterial symbiont of phloem sap-feeding insects with a genome size of only 160 kbp [9]. Others, like the endosymbionts of the rice weevils *Sitophilus zeamais *(SZPE) and *Sitophilus oryzae *(SOPE) as well as *Sodalis glossinidius*, a symbiont of tsetse flies [10-12] represent the other extreme, and exhibit only slight reduction in genome size in comparison to free-living close relatives. Unlike the genomes of *Buchnera *and *Carsonella*, the genomes of these endosymbionts do not exhibit unusually high AT content.

Intracellular symbionts including SZPE and SOPE, have lost at least one of the recombinational repair enzyme genes encoded by *rec*A and *rec*F, a characteristic found in all other bacterial intracellular symbionts. The only known exception to this finding is the observation of intact genes for *rec*A and *rec*F in *S. glossinidius*. The occurrence of genes for flagella apparatus still encoded in the genome of *S. glossinidus *might indicate that this symbiosis has only recently been established [12]. Cyanobacterial interactions with plants and protists are also well known [13-17]. These associations are in most cases facultative, and do not involve vertical transmission. In these cases, the cyanobacterial symbiont re-infects the host every generation. As with other facultative symbioses, genetic modification of the endosymbiont genome is yet undetected [17].

The pennate diatom *Rhopalodia gibba *harbours endosymbionts closely related to extant cyanobacteria. Some of the closest free-living relatives of these so-called spheroid bodies are diazotrophic cyanobacteria of the *Cyanothece *sp. group [18]. The spheroid bodies encode genes for nitrogen fixation and have the capacity to fix molecular nitrogen [18,19]. Although the spheroid bodies are of cyanobacterial origin, they lack the typical photosynthetic pigmentation; and thus have been assumed to be photosynthetically inactive. Unlike all other unicellular nitrogen fixing cyanobacteria, they fix nitrogen under light conditions only [18-20]. We observe one to four spheroid bodies per host cell depending on culture conditions which are transmitted vertically to the daughter cells during host cell division [15]. Altogether, these findings have led to the supposition that the spheroid bodies of *R. gibba *are obligate endosymbionts. Physiological adaptation to an intracellular endosymbiotic association is expected to result in genome modification and this expectation has motivated our investigation of gene structure, content and organisation in the spheroid body's genome of *R. gibba*. In order to investigate this, we have constructed fosmid libraries of the spheroid body and *Cyanothece *sp. ATCC 51142 and analysed genomic regions of special interest. Here we describe observations and analyses of the *nif*-gene region and also loci relevant to the question of the obligate nature of the spheroid body endosymbiosis. Our investigations show massive genomic changes introduced into the spheroid body's genome. These include inactivation and losses of genes and the creation of large non-coding AT-rich areas. Our observations confirm the obligate nature of the spheroid body endosymbiont and provide insight into the nature of genome changes that accompany endosymbiosis and organelle formation.

## Results

### The *nif*-gene region of spheroid bodies and *Cyanothece *sp. ATCC 51142

**Figure 1:**
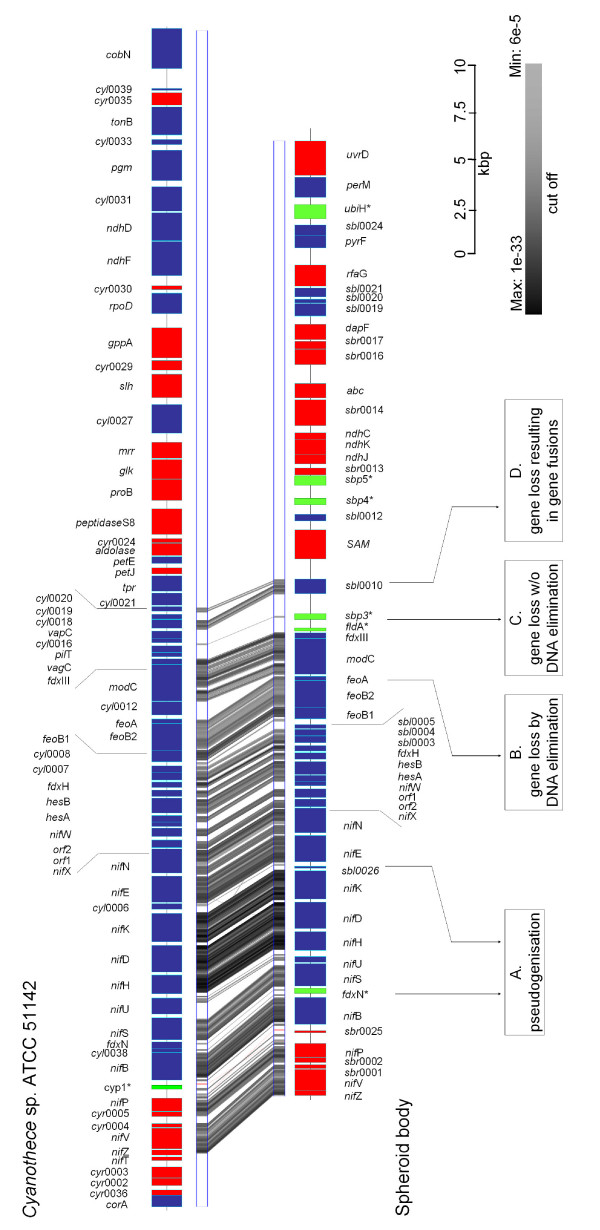
**Gene content in, and downstream of, the *nif *gene region of *Cyanothece *sp. ATCC 51142 and spheroid body of *R. gibba***. Blue and red bars represent *orfs *coded on the leading or lagging strand of DNA, respectively. The locations of pseudogenes in the spheroid body fragment have been indicated with green bars. Genes have been named either according to homology matches in BLAST analyses or numbered consecutively for each organism (see also additional files 1 and 2). A GATA [29] plot is shown and indicates regions of high synteny between both organisms. GATaligner settings were: Window size: 100; Match: 5; MisMatch:-4; Gap Creation:-10; Gap Extension:-4; Raw Score Cut Off: 80. GATAPlotter score settings: Max: 141 bits, expect 1E-33; Min: 46.8 bits, expect 5E-5. GATAPlotter scores have been represented using a greyscale bar. Regions of the spheroid body genome showing modifications of special interest have been indicated. A) Gene inactivation by pseudogenisation (e.g. *fdx*N*); B) Gene deletion with DNA loss (e.g. *cyl*0012); C) Gene deletion without DNA loss resulting in large non-coding regions (e.g. *cyl*0016); D) Gene deletion with DNA loss resulting in gene fusion (e.g. *cyl*0019). See text for further description of individual modifications.

**Figure 2:**
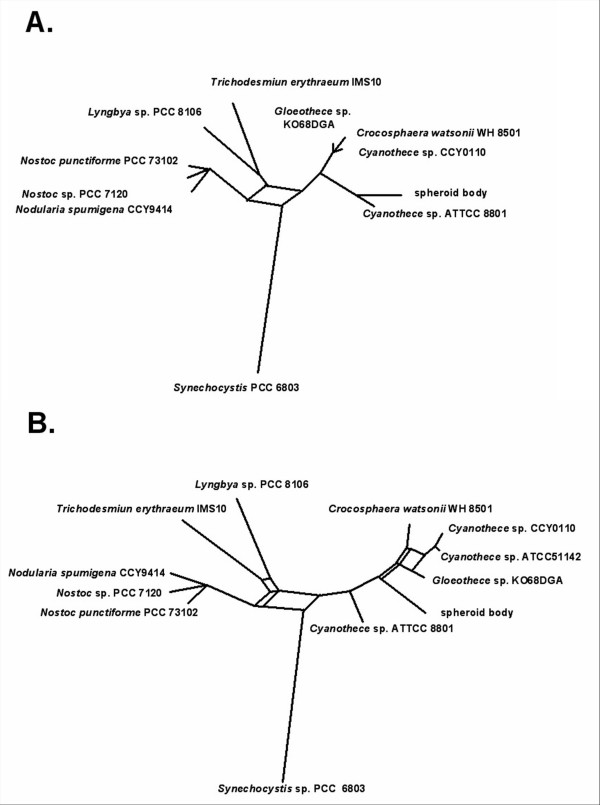
**Supernetworks displaying relationships inferred from phylogenetic analysis of different gene regions**. **A**. Supernetwork reconstructed using strict consensus maximum likelihood trees for NifB, NifN, NifS and ABC, HesA, NdhK, ModC, PerM, UvrD, SAM, Sbr0014 of different cyanobacetria (see Materials and Methods). **B**. Supernetwork reconstructed using strict consensus maximum likelihood trees for NifD, NifH, NifK, NifE, and ABC, HesA, NdhK, ModC, PerM, UvrD, SAM, Sbr0014. The networks are outgroup rooted using *Synechocystis *PCC 6803. A reticulation occurs in the centre of both graphs because of the local instability in the placement of *Synechocystis *PCC 6803.

For this study we cloned and sequenced the *nif*-operon and flanking regions from the genomes of the intracellular symbiont of the diatom *R. gibba*, the spheroid bodies, and a close free-living relative, the diazotrophic cyanobacterium *Cyanothec*e sp. ATCC 51142 (Figure 1). Altogether we sequenced and analysed a contiguous 63,362 bp *Cyanothece *fragment comprising the *nif *gene region and a contiguous 51,475 bp fragment including the corresponding region of the spheroid body's genome. Additionally, we sequenced and analysed 140,000 bp of non-contiguous genomic DNA of the endosymbiont. Using these additional datasets we characterised sequences in the spheroid body's genome for *rec*A, *rec*F, *psb*C and *psb*D. For further analyses we also used information from the genome of the recently sequenced *Cyanothece *strain CCY0110 and other closely related cyanobacteria whose genomes have been sequenced and are available in the NCBI nr database. To obtain a phylogenetic framework for making inference of genome modification in the spheroid body's genome we reconstructed maximum likelihood gene trees for all homologues greater than 200 amino acids in the 63,362 bp region. Where possible, trees were outgroup rooted using homologues from *Synechocystis *sp. PCC 6803. Figure 2 shows supernetworks [21] built for these taxa. These networks summarise the relationships in individual gene trees which do not necessarily need to have the same taxon sampling. In this analysis, with some proteins (NifB, NifN, NifS), the spheroid body's genome was found to be most closely related to *Cyanothece *sp. ATCC 8801 (Figure 2a) but with other proteins (NifD, NifH, NifK, NifE), spheroid body sequences have a closer phylogenetic relationship with *Cyanothece *sp ATCC 51142, *Cyanothece *strain CCY0110, *Crocosphaera watsonii *WH 8501, and *Gloeothece *sp. KO68DGA (Figure 2b).

Figure 1 summarises the *nif*-operon-related regions of *Cyanothece *sp ATCC 51142 and the spheroid body's genome. In both, components of the nitrogenase dependent cyanobacterial nitrogen fixation machinery are encoded, including structural genes of the nitrogenase *nif*H, *nif*D and *nif*K, cofactors (*nif*B, *nif*N, *nif*E, *nif*V, *nif*W) and processing proteins for metal centre biosynthesis (*nif*U, *nif*S). As shown in figure 1, synteny of the *nif*-genes including the size of intergenic regions is very high. There is an overall G/C-content of 40.8% for this region of *Cyanothece *genome and a G/C-content of 37.2% for the spheroid body sequence. Codon usage is nearly equivalent in both genome regions with a slight AT-bias at the third codon position in the spheroid body genes (see Table 1).

**Table 1:** Genome features of obligate intracellular symbiotic and parasitic bacteria

Species	Genome size (Mbp)	AT-content genome (%)	AT-content 16S rDNA (%)	ATcontent 1st codon	ATcontent 2nd codon	ATcontent 3rd codon	Ref.
*Buchnera aphidicola *APS	0.66	73.7	49.86	62.62	69.41	85.77	[39]
*Candidatus Blochmannia floridanus*	0.71	72.6	52.89	61.33	68.08	83.95	[7]
*Carsonella ruddii*	0.16	83.4	64.15	80.26	79.61	92.22	[9]
*Chlamydia trachomatis *D/UW-3/CX	1.04	58.7	48.58	48.39	61.17	65.52	[40]
*Rickettsia prowazekii *Madrid E	1.11	71.0	49.5	58.95	68.24	81.53	[22]
*Sodalis glossinidius*	4.17	45.3	45.29	38.83	57.86	34.73	[12]
*Wigglesworthia glossinidia*	0.7	77.5	51.19	69.12	71.84	88.12	[38]
**Spheroid body**	**n.d**.	**62.8 (fragment)**	**45.77**	**48.16**	**61.89**	**67.25**	**this paper**
*Cyanothece sp. ATCC 51142*	n.d.	60.2 (fragment)	45.42	49.16	61.40	66.81	this paper

Genome size, AT-content and nucleotide composition of each codon position are indicated

(References: [7,9,12,22,38–40]). n.d.: not determined

**Figure 3:**
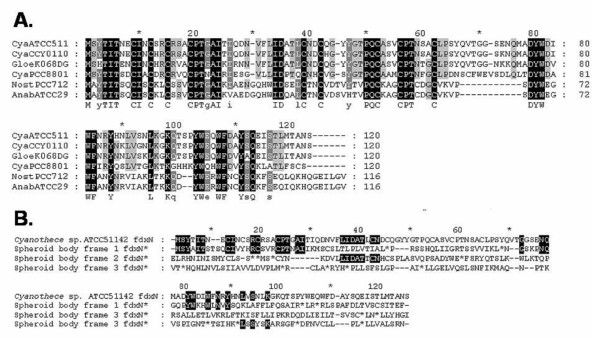
**Analysis of Ferredoxin N**. **A**. Multiple alignment of cyanobacterial FdxN proteins. Accession numbers: CyaATCC51142: AAW56985.1 (*Cyanothece *sp. ATVV51142); CyaPCC8801: AAC33373.1 (*Cyanothece *sp. PCC8801); CyaCCY0110: ZP_01727762.1 (*Cyanothece *sp. CCY0110); GloeK068DGA: BAF47148.1 (*Gloeothece *sp. KO68DGA); NostPCC7120: AAA22005.1 (*Nostoc punctiforme *PCC 7120); AnabATCC29413: YP_324413.1 (*Anabaena variabilis *ATCC 29413). **B**. Alignment of *Cyanothece *sp. ATCC51142 FdxN protein with the spheroid body fdxN* pseudogene translated in 3 forward reading frames. Evidence of homology, at the level of amino acid similarity, is distributed across all 3 reading frames of the pseudogene, indicating multiple substitutions and single nucleotide deletion events.

**Figure 4:**
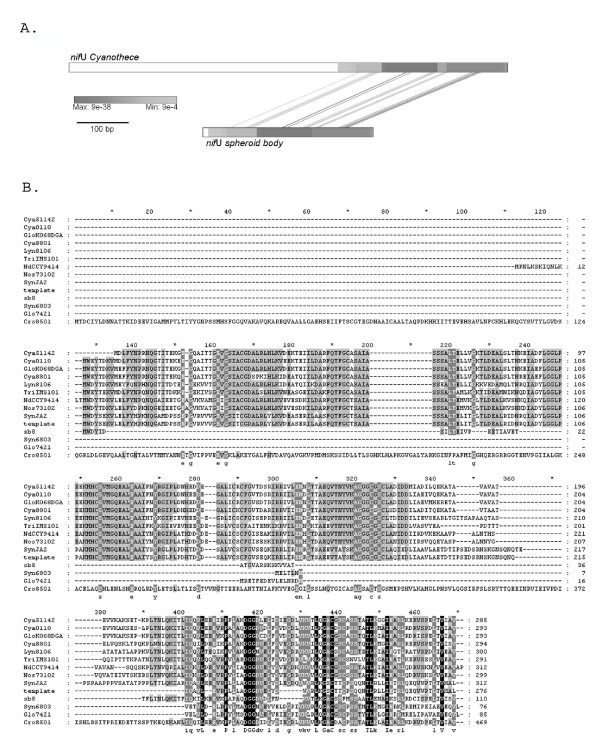
**Truncation of *nif*U in spheroid bodies**. **A**. GATA plot for *Cyanothece *and spheroid body *nif*U indicating conserved regions. GATaligner settings: Window size: 100; Match: 5; MisMatch:-4; Gap Creation:-10; Gap Extension:-4; Raw Score Cut Off: 92.0. GATAPlotter score settings: Max: 141 bits, expect 9E-38; Min: 28 bits, expect 9E-4. GATAPlotter scores are indicated. **B**. Multiple alignment of predicted amino acid sequences for NifU indicating an N-terminal truncation in the homologue from the endosymbiont. NifU accession numbers: Cya51142: AAW56987.1 (*Cyanothece *sp. ATCC 51142); Cya0110: ZP_01727764.1 (*Cyanothece *sp. CCY0110); GloKO68DGA: BAF47150.1 (*Gloeothece *sp. KO68DGA); Cya8801: AAC33371.1 (*Cyanothece *sp. PCC 8801); Lyn8106: ZP_01620769.1 (*Lyngbya *sp. PCC 8106); TriIMS101: AAF82636.1 (*Trichodesmium *sp. IMS101); NdCCY9414: ZP_01628437.1 (*Nodularia spumigena *CCY9414); Nos73102: ZP_00112317.1 (*Nostoc punctiforme *PCC 73102); SynJA2: YP_476679.1 (*Synechococcus *sp. JA-2-3B'a(2–13)); sb8: AAW57048.1 (spheroid body); Syn6803: NP_442853.1 (*Synechocystis *sp. PCC6803); Glo7421: NP_925823.1 (*Gloeobacter violaceus *PCC 7421); Cro8501: ZP_00516385.1 (*Crocosphaera watsonii *WH 8501).

Although the spheroid body's genome contains the same set of genes at the *nif *locus as in *Cyanothece *ATCC 51142, remarkable differences are apparent. Notably, between *nif*B and *nif*S, the functional *fdx*N gene is replaced by a pseudogene (*fdx*N*) in the spheroid body's genome. The coding sequence is interrupted by several stop-codons. In contrast, an intact reading frame for *fdx*N is conserved in all other close free-living cyanobacterial relatives, indicating formation of the pseudogene is a derived feature of the spheroid body lineage (Figure 3). The presence of a truncated *nif*U gene is also derived on the endosymbiont lineage, corresponding to approximately 170 amino acids of the N-terminus (Figure 4). This *nif*U homologue still encodes an intact open reading frame for the [2Fe-2S] binding and C-terminal NifU-domain. Interesting, in more distantly related cyanobacteria (*Synechocystis *sp. PCC 6803 and *Gloeobacter violaceus *PCC 7421) a truncated homologue is also present. Based on the phylogenetic analyses reported in Figure 2 and also comparative analyses of the NifU protein (not shown) it appears that truncation of *nif*U in spheroid bodies is a derived feature of the endosymbiont lineage.

### Gene deletions and modifications downstream the *nif*-region

Conserved downstream of the *nif*-operon genes in *Cyanothece *ATCC 51142 and the spheroid body are genes encoding subunits of the NADH-dehydrogenase and ferredoxin as well as transporters for Fe and Mo (Figure 1). However, genes encoding photosynthetic proteins, which are precursors for cytochrome c6 (*pet*J) and plastocyanin (*pet*E), are absent in the spheroid body in this genome region. Interestingly, elsewhere in the genome, the photosystem II protein genes *psb*C (CP43) and *psb*D (D2) exist in an operon-like structure, similar to that of *Cyanothece *sp. CCY0110 and other cyanobacteria. However, in the spheroid body these genes are highly truncated or disrupted by several stop codons and thus exist as pseudogenes. This finding is consistent with the lack of photosynthetic activity previously reported for the spheroid body [18,19].

**Figure 5:**
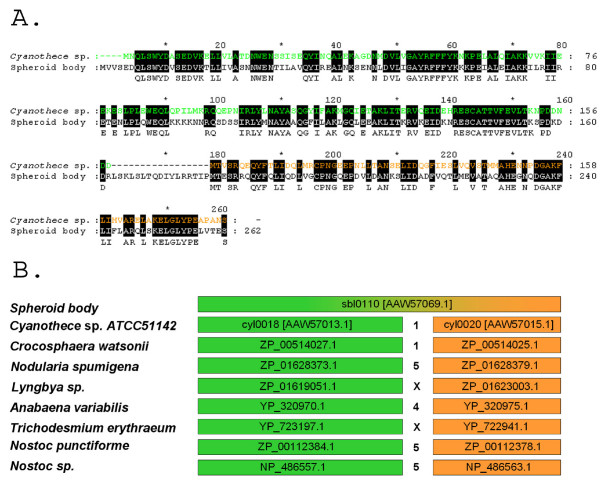
**Spheroid body *orf sbl*0010 encodes a fusion protein derived from homologues of the *Cyanothece *sp. ATCC51142 Cyl0018 and Cyl0020 proteins**. **A**. Alignment of predicted amino acid sequences for spheroid body protein Sbl0010 and *Cyanothece *proteins Cyl0018 and Cyl0020. Deletion in the endosymbiont genome of *cyl*0019 in the creation of *sbl*0010 can be inferred during reductive genome evolution. In Sb10010, homologues of Cyl0018 and Cyl0020 have been conserved in full length and are separated by a 17 amino acid residues. Cyl0018: green, Cyl0020: orange, Sbl0010: black. **B**. Cyl0018 and Cyl0020 are highly conserved in cyanobacteria closely related to the spheroid body. They are aseparated by 1–5 genes when they co-occur at the same locus, but in some cases they are encoded at different loci of the genome (indicated by x).

Downstream of the highly conserved *nif*-gene region, other genetic modifications can be inferred. In *Cyanothece *ATCC 51142, and other closely related cyanobacteria the open reading frame (*orf*)*cyl*0012 is flanked by the genes for an iron-transporter (*feo*A) and a Mo-ABC-transporter (*mod*C). This orf has been deleted in the spheroid body's genome (Figure 1). In this case the whole gene has been removed without trace of pseudogenisation or local sequence conservation. The *orf *identified as *cyl*0019 in *Cyanothece *ATCC 51142 has also been lost from the *nif*-region. In this species and other close relatives, this *orf *is flanked by two conserved *orfs cyl*0018 and *cyl*0020. In the spheroid body's genome, *cyl*0019 is deleted and the flanking *orfs cyl*0018 and *cyl*0020 are fused to give to the hypothetical protein *sbl*0010 (Figure 5). Not all deletions of genes in the spheroid body's genome have resulted in genome compaction as some genome regions appear replaced by non-coding regions as described in the following section.

### Extensive modifications lead to large non-coding regions in the spheroid body's genome

**Figure 6:**
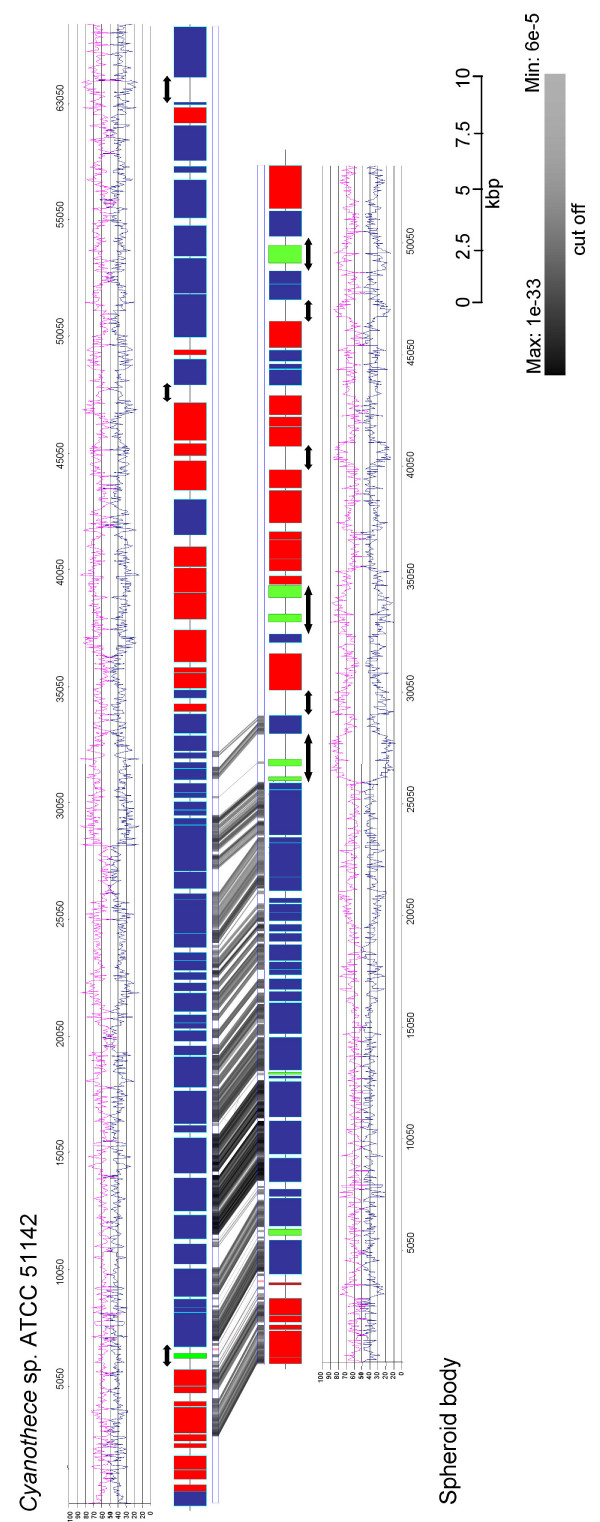
**A/T-G/C frequencies in *Cyanothece *sp. ATCC 51142 and spheroid body genome fragments**. A/T-G/C-plot for the genome region shown in Fig. 1 (red: AT; blue:G/C), indicating a high AT composition in the large non-coding regions (black arrows) of the spheroid body fragment.

A significant difference between the genome of *Cyanothece *sp. ATCC 51142 and that of the spheroid body is the extent of non-coding DNA stretches greater than 500 bp (Figure 6). In the former there are three non-coding stretches at the *nif *locus (including downstream region). There are seven such regions in the spheroid body's genome fragment. One of these additional non-coding regions is located adjacent to the *nif *gene region, in a region of high synteny between both genomes. The non-coding regions of the spheroid body's genome are characterised by elevated levels of A and T nucleotides. Several pseudogenes are also located within these genome regions (Figure 1 and 6).

In *Cyanothece *ATCC 51142, the gene *fdx*III and an *orf *coding for a conserved hypothetical protein (*cyl*0018) flank four open reading frames (*cyl*0014/*vag*C, *cyl*0015/*pil*T, *cyl*0016, *cyl*0017/*vap*C). Three of these have highest similarity to *virulence associated proteins *(vap-proteins) and the fourth has greatest similarity with proteins containing a PIN-domain (Figure 1). In spheroid bodies, *fdx*III and the homologue to *cyl*0018 (as part of *sbl*0010, see above) frame a non-coding region of about 2000 bp which contains two pseudogenes: for a flavodoxin, long-chain hypothetical protein and an *orf *conserved in *Crocosphaera *sp.(CwatDRAFT_1967). This non coding region shows an increased AT-ratio of 73.5% (Figure 6). Its presence is the result of extensive genome modification which has led to the deletion of multiple genes. Unlike other regions where there has been gene deletion (e.g. as with *cyl*0012), this modification is not accompanied by the deletion of the genomic regions. It is possible that these regions might be the outcome of multiple mutations leading to the loss of genes, no longer recognizable as pseudogenes, with preservation of the genomic locus, which might be subsequently eliminated [22]. Because the whole genome size of spheroid bodies is not experimentally determined so far, it is not known if these modifications have lead to an overall decrease of the spheroid body genome size. As described in more detail in the discussion part, we used a bioinformatic model to predict the size of the whole spheroid body genome. Using this prediction, the genome size is estimated to be approximately 2,6 Mb.

**Figure 7:**
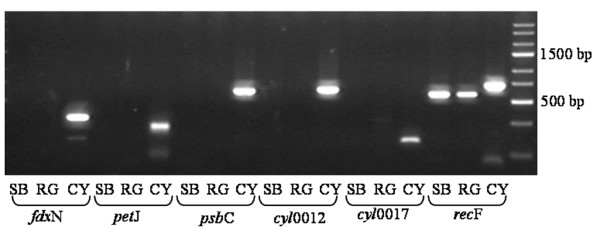
**PCR analysis of missing or pseudogenisised genes**. PCR analysis of *Cyanothece *sp. ATCC 51142 (CY), *R. gibba *(RG) and spheroid body (SB) DNA. Amplification with primers specific for the cyanobacterial *fdx*N, *pet*J, *psb*C, *cyl*0012 and *cyl*0017 genes show that the analysed genes are not encoded in *R. gibba *and the endosymbiont's genome. CYrecF and SBrecF were used as positive controls. GeneRuler™ Express DNA Ladder (Fermentas) was used as molecular weight standard.

To investigate whether intact copies of missing or pseudogenised genes (e.g. after genome rearrangement or gene duplication events) are present elsewhere in the spheroid body's genome, we performed PCR analysis with specific or degenerate primers for the cyanobacterial genes *fdx*N, *pet*J, *psb*C, *cyl*0012 and *cyl*0017. As shown in Figure 7, no products were amplified using either spheroid body or *R. gibba *DNA as template, indicating that the identified (pseudo)genes do not have functional counter parts encoded elsewhere in the endosymbiont's genome.

### RecA and RecF are encoded by the spheroid body's genome

**Figure 8:**
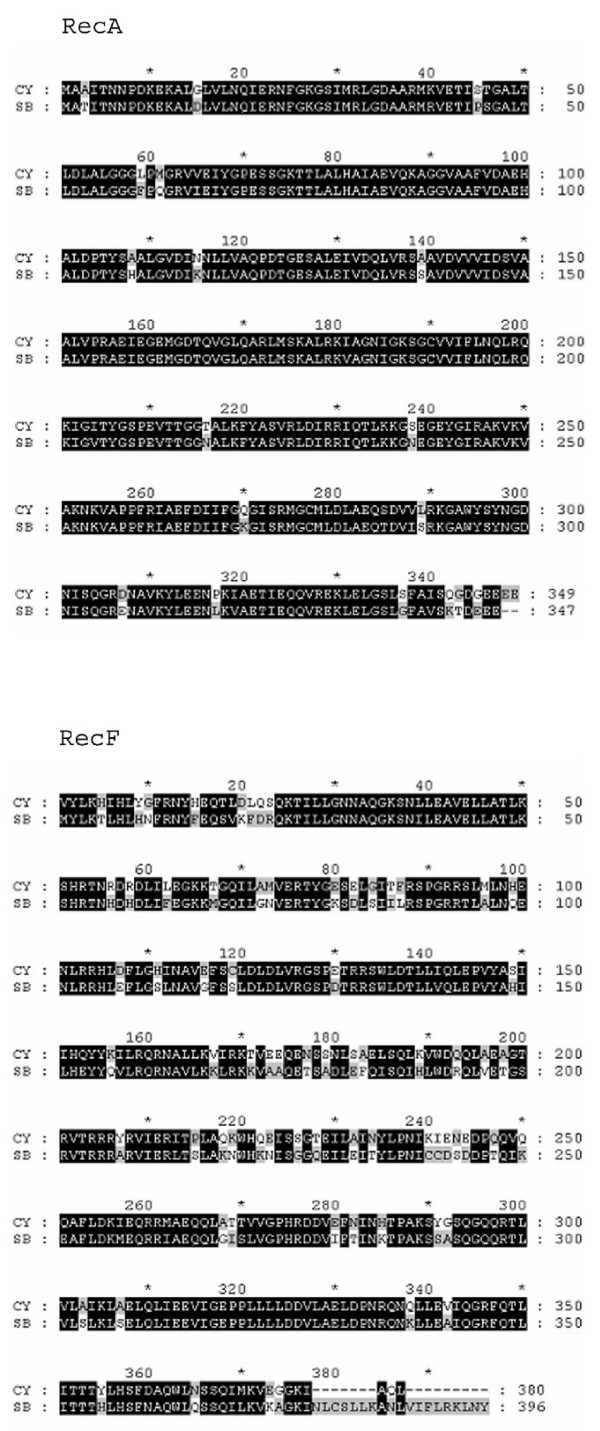
**Pairwise alignments for RecA and for RecF**. Alignment of RecA (left) and RecF (right) homologues from the spheroid body (SB) and *Cyanothece sp*. ATCC51142 (CY) genomes. The spheroid body encodes complete full length *rec*A and *rec*F *orfs*.

The proteins RecA and RecF play an important role in recombinational repair of DNA as well as roles in other repair pathways like nucleotide excision (reviewed in [23]). From the study of insect-bacterium symbioses it is known that gene loss and pseudogene creation is often associated with defects in *rec*A, and/or *rec*F [10]. In order to investigate whether the observed inactivation and deletion of genes in the spheroid body's genome might be explained by defects in the bacterial repair systems, we searched the genomes of the spheroid body and of *Cyanothece *ATCC 51142 for *rec*A and *rec*F. As shown in Figure 8, intact and highly conserved *orfs *can be identified in both genomes, indicating that repair pathways are active in the *R. gibba *endosymbiont.

### Accumulation of pseudogenes in the spheroid body's genome

As already mentioned, by analysing the contiguous spheroid body's genome fragment (Figure 1) several pseudogenes could be readily identified, among them a mutated ferredoxin gene (*fdx*N*) within the *nif*-operon as well as other genes downstream of the *nif*-gene region. Additional pseudogenes for conserved cyanobacterial proteins were also identified when screening 140,000 bp of non-contiguous genome sequences from the spheroid bodies. These were found either via BLAST homology search (minimum e-value: 5e-10) or by analysis of spheroid body's genome regions corresponding to operons conserved in other cyanobacteria. Genetic regions with homologues in other species, divided into two fragments by one stopcodon or frameshift or just truncated were considered pseudogenes if the similar gene region was less than half the length of its homologue [12]. Deleterious mutations leading to pseudogenes were found in genes encoding proteins that affect cellular processes such as cell wall biosynthesis and transposon controlled genome rearrangement (summarized in Table 2). In *Cyanothece *sp. ATCC 51142, only one pseudogene was identified within the genome fragment that contained the *nif-*operon (Table 2). In contrast, six pseudogenes were found in the spheroid body's genome fragment. No pseudogenes were identified in the genome of *Cyanothece *sp. strain CCY0110 which is a very close relative of *Cyanothece *sp. ATCC 51142.

**Table 2:** Pseudogenes in all sequenced spheroid body genome fragments (totalling 192335 bp) and pseudogenes identified in the *Cyanothece *sp. ATCC 51142 genome region shown in Figure 1 (63344 bp)

**No**.	Name	Best BlastX hit	Organism	Accession	e-value
sbp1*	*fdx*N*	FdxN	*Cyanothece *sp. PCC 8801	AAC33373.1	0.070
sbp2*	*fld*A*	Flavodoxin, long chain	*Crocosphaera watsonii *WH 8501	ZP_00515759.1	2e-05
sbp3*	*sbp*3*	hypothetical protein CwatDRAFT_1967	*Crocosphaera watsonii *WH 8501	ZP_00517249.1	7e-05
sbp4*	*sbp*4*	hemolysin	*Cyanothece *sp. CCY0110	ZP_01728583.1	3e-09
sbp5*	*sbp*5*	Putative esterase	*Crocosphaera watsonii *WH 8501	ZP_00514941.1	6e-16
sbp6*	*ubi*H*	COG0654: 2-polyprenyl-6-methoxyphenol hydroxylase and related FAD-dependent oxidoreductases	*Nostoc punctiforme *PCC 73102	ZP_00110350.1	2e-17
sbp7*	*sbp*7*	hypothetical protein CY0110_23906	*Cyanothece *sp. CCY0110	ZP_01727952.1	5e-09
sbp8*	*sbp*8*	hypothetical protein CY0110_11227	*Cyanothece *sp. CCY0110	ZP_01730915.1	1e-18
sbp9*	*sbp*9*	Peptidase S49, SppA 67 kDa type:Peptidase S49, SppA	*Crocosphaera watsonii *WH 8501	ZP_00518433.1	3e-09
sbp10*	*sbp*10*	GDP-D-mannose dehydratase	*Cyanothece *sp. CCY0110	ZP_01730804.1	1e-131
sbp11*	*sbp*11*	NAD-dependent epimerase/dehydratase	*Crocosphaera watsonii *WH 8501	ZP_00513970.1	5e-103
sbp12*	*sbp*12*	hypothetical protein CwatDRAFT_2668	*Crocosphaera watsonii *WH 8501	ZP_00517115.1	3e-10
sbp13*	*uma*4*	Uma4 (transposase homolog)	*Microcystis aeruginosa *PCC 7806	AF183408_15	4e-05
sbp14*	*sbp*14*	hypothetical protein CY0110_27355	*Cyanothece *sp. CCY0110	ZP_01729498.1	9e-07
sbp15*	*sbp*15*	thioredoxin reductase	*Lyngbya *sp. PCC 8106	ZP_01622501.1	8e-88
sbp16*	*sbp*16*	putative transposase	*Lyngbya *sp. PCC 8106	ZP_01618921.1	6e-65
sbp17*	*lld*P*	hypothetical protein DSY2261	*Desulfitobacterium hafniense *Y51	YP_518494.1	1e-14
sbp18*	*sbp*19*	Mg chelatase-related protein	*Cyanothece *sp. CCY0110	ZP_01732243.1	2e-06
sbp19*	*sbp*20*	hypothetical protein CY0110_07314	*Cyanothece *sp. CCY0110	ZP_01728735.1	2e-103
sbp20*	*rsb*W*	Putative Anti-Sigma regulatory factor (Ser/Thr protein kinase)	Cyanothece sp. CCY0110	ZP_01726436.1	0.011
sbp21*	*sbp*22*	Anti-Sigma-factor antagonist (STAS) and sugar transfersase	*Cyanothece *sp. CCY0110	ZP_01726435.1	7e-53
sbp22*	*sbp*23*	transposase	*Nostoc *sp. PCC 7120	NP_484634.1	4e-20
sbp23*	IRK*	hypothetical protein CY0110_12822	*Cyanothece *sp. CCY0110	ZP_01729432.1	5e-28
sbp24*	*orf*AB*	orfAB	*Nostoc *sp. PCC 7120	AAC97588.1	3e-05
sbp25*	*sbp*26*	Protein of unknown function DUF820	*Crocosphaera watsonii *WH 8501	ZP_00514570.1	2e-13
sbp26*	*sbp*27*	probable sulfotransferase	*Cyanothece *sp. CCY0110	ZP_01732095.1	4e-50
sbp27*	*sbp*28*	COG3464: Transposase and inactivated derivatives	Nostoc punctiforme PCC 73102	ZP_00108066.1	2e-11
sbp28*	*s*_TKc*	serine/threonine protein kinase	*Cyanothece *sp. CCY0110	ZP_01731251.1	4e-26
sbp29*	*sbp*30*	hypothetical protein CwatDRAFT_4770	*Crocosphaera watsonii *WH 8501	ZP_00515299.1	8e-07
sbp30*	*ded*A*	DedA	*Lyngbya *sp. PCC 8106	ZP_01623614.1	2e-53
sbp31*	*sbp*32*	hypothetical protein CY0110_03639	*Cyanothece *sp. CCY0110	ZP_01727528.1	5e-06
sbp32*	*mel*B *	Sodium:galactoside symporter	*Crocosphaera watsonii *WH 8501	ZP_00517271.1	5e-22
sbp33*	*psb*C*	photosystem II CP43 protein	*Synechocystis *sp. PCC 6803	NP_441119.1	1,0
sbp34*	*psb*D*	photosystem II D2 protein	*Cyanothece *sp. CCY0110	ZP_01728138.1	5e-05
cyp1*	*cyp*1*	hypothetical protein CY0110_22382	Cyanothece sp. CCY0110	ZP_01727759.1	2e-11

Pseudogenes within the *nif*-operon and downstream regions (genome regions shown in Figure 1) are indicated in grey.

## Discussion

An intriguing example of an obligate intracellular symbiotic interaction is the cyanobacterium-diatom symbiosis found in *Rhopalodia gibba *[18]. Here the symbiont (spheroid body) can fix nitrogen for its eukaryotic host, and we have hypothesised that this capacity has been a driving force for establishing the intracellular endosymbiotic relationship [17]. The spheroid body of *Rhopalodia gibba *provides an opportunity to investigate changes in endosymbiont physiology and genome evolution during adaptation of a symbiont to an intracellular environment.

Previous studies have reported changes in the genomes of bacteria following development of symbiotic relationships. In bacteria that are thought to have recently or transiently become symbiotic, changes include occurrence of multiple transposable elements and deletions of important components of recombinational DNA repair mechanisms [1]. In longer established symbiotic and parasitic eukaryote-bacterium interactions, significant gene losses have been observed, and these have been accompanied by reduction of genome size and generation of AT rich genomes [3,6,24]. Changes that have occurred in the spheroid body's genome can not be categorised as an obvious example of the former or latter relationship. For example, the spheroid body's genome encodes several transposase genes, all with disrupted reading frames, indicating that these are pseudogenes. This finding is consistent with stability of the diatom-spheroid body endosymbiosis and a long term host-endosymbiont interaction, which can be traced back to the Miocene [25]. Contrasting with the occurrence of transposase pseudogenes is evidence suggesting a functional DNA repair system in the spheroid body's genome. This is a finding more consistent with a relatively young endosymbiotic relationship. In nearly all intracellular bacteria studied to date, at least one of the genes encoding the DNA repair proteins RecA and RecF has been eliminated. It is thought this might be necessary to facilitate restructuring of the symbiont genome (for the exception see [12]). In the spheroid body's genome both *rec*A and *rec*F are present and have intact open reading frames. Thus the genome modifications that we report for the spheroid body's genome have all occurred against a background of a presumably intact DNA repair system. These modifications suggest that selective pressure for certain genes has changed upon establishment of the interaction, and the challenge is to attempt to understand the potential relevance of these for necessary and redundant functions in an obligate endosymbiotic relationship. For example, gene truncations as detected e.g. in *nif*U, would remove genes redundant for diazotrophic growth [26], and such deletion might be an early event in genome reduction of the symbiont. A subsequent or perhaps parallel step would include the inactivation of genes whose gene products are no longer needed for the initial symbiotic association. For this to occur, various different possible scenarios could be hypothesized: inactivation of genes by deleterious mutations resulting in the accumulation of pseudogenes or loss of genome fragments by deletion of larger DNA portions via rearrangements [27]. Another hypothesis posits a "domino-effect" of initial pseudogenisation triggering subsequent large-scale gene loss [28]. In this scenario, random pseudogenisation might lead to the inactivation of a pathway due to mutation of a single essential factor, followed by large-scale deletion of other genes involved in this pathway. In each case, the selective pressure would be different for genes coding for different functions, and loss would depend on whether function could be compensated by other genes in the endosymbiont or host cell genome. In the latter case, as in highly adapted interactions, signal-dependent transport of the protein from the host cytoplasm to the endosymbiont would be necessary.

We detected several examples for the disruption of coding regions by mutations (Table 1), in which the original gene is still detectable by analysis of all three possible frames. This includes psuedogenisation of *fdx*N (*fdx*N*), a gene which has been found to be non essential for nitrogen fixation in *Anabaena variablis *[20] and several other genes on spheroid body's genome fragments that we have sequenced (Table 2, Figure 1). Such observations provide further evidence that pseudogenisation of genes, which are non-essential for endosymbiotic life-style, is an important feature in the early reductive genome evolution of obligate intracellular cyanobacteria. Gene loss through independent DNA deletion events could also be inferred in comparative analysis of the spheroid body's genome fragment; among these the deletion of factors conserved in diverse cyanobacterial lineages (*cyl*0012, *cyl*0019). Due to elimination of the immediate DNA region, these modifications have led to a localised increase in gene density. In one extreme, deletion has produced a fusion of non-adjacent genes on the endosymbionts genome (*sbl*0010). In other cases of gene deletion, genes have been removed and replaced with non-coding sequence that is much higher in AT-content than occurs in the coding regions (Figure 6). It is unclear whether this difference in composition reflects a shift in substitutional bias favouring A and T residues, and/or whether an existing bias becomes more apparent in de-novo regions that are under reduced structural/function constraint. In either event, the existence of these AT rich non-coding regions suggests that pseudogenisation and DNA deletion are not inevitably linked events in a sequential process of degenerative genome evolution in spheroid bodies. However, non-coding regions are rare in genomes of free-living bacteria. Since DNA can be introduced in several ways into prokaryotic genomes, their compactness is maintained by the deletion of harmful DNA. Given the intracellular existence of spheroid bodies, it is possible that their genome is less exposed and less susceptible to introductions of foreign DNA through mechanisms of horizontal gene transfer and lysogenic bacteriphages in comparison to those of free-living bacteria. If so, processes excluding non-coding DNA and pseudogenes from the spheroid body's genome may well be less efficient than those operating in free living bacteria. Such a hypothesis might help explain the greater extent of non-coding DNA and pseudogenised genes in the spheroid body's genome. Increased mutation rates, thought to be associated with reductive genome evolution would contribute to accumulation of these genome features [29]. The genome modifications observed in the spheroid body are in some respects comparable to those of *Sodalis glossinidius*. A large fraction (49%) of the *Sodalis *genome is composed of non-coding DNA that has accompanied reductive genome evolution. Moreover, the *Sodalis *chromosome contains many unusual pseudogenes [12]. The spheroid body's genome differs from *Sodalis *with respect to their generally higher AT-content.

The diverse features of reductive genome evolution in obligate intracellular symbionts (and pathogens) include a significant reduction of overall genome size in these organisms. However, the experimental determination of the spheroid body's genome size using standard molecular techniques is difficult due to the extreme stability of the host-spheroid body interaction and the limited amount of intact and purified endosymbionts that can be obtained from *R.gibba*. Recently in a study on the dynamics of reductive evolution, exponential relationships were inferred between genome size and SSU rDNA GC-content in mitochondria, free-living and obligate intracellular bacteria [30]. Based on the model these authors propose, and using 16S sequence data previously published [18], we have estimated that the genome size of spheroid bodies is approximately 2.6 Mb. The genome size of free-living *Cyanothece *sp. CCY0110 is 5.8 Mb. Hence if our estimate of the spheroid body's genome size is accurate, this estimate suggests that reduction has produced a genome currently similar in size to that of *Synechococcus *(2.2–2.6 Mb), and may indicate that the endosymbiosis is still at an early state of development.

Our comparative analyses of spheroid body's genome fosmid sequences indicate that the photosynthetic genes *psb*C and *psb*D have been inactivated by mutation in the endosymbiont genome. These gene products are essential factors in the photosynthetic light reaction of photosystem II [31]. According to the "domino-effect" hypothesis [28] initial deletion of components such as PsbC and PsbD is expected to lead to mass deletion of other genes involved in photosynthetic light reactions. Consistent with this prediction, additional photosynthetic factors that occur in closely related cyanobacteria are either absent (e.g. the cytochrome PetJ and the plastocyanine precursor PetE) or appear as a non functional pseudogene (e.g. the flavodoxin *fld*A*) in the spheroid body's genome.

Aside from gene loss resulting from reductive genome evolution, the absence of certain genes within the analysed genome region could also be explained by gene duplications or rearrangements. Without the complete sequence of the spheroid body's genome we can not exclude the possibility that following duplication, pseudogenisation has affected copies of some genes within the analysed genome region, while functional copies are retained elsewhere. However, the phenotypic loss of photosynthetic pigmentation indicates a complete loss of at least one essential factor of photosynthesis in the endosymbiont's genome. In addition, PCR analysis did not identify intact *psbC *and *psbD *genes present anywhere else in the spheroid body's genome (Figure 7).

The diverse modifications in the analysed spheroid body's genome fragment are not equally distributed over the whole sequence but accumulate downstream of the conserved *nif *gene region (Figure 1 and 5). This skewed distribution of degenerative modifications possibly reflects purifying selection acting across this genome region during the molecular adaptation process [32]. Aside from the mutation of *fdx*N* – a protein unimportant for nitrogen fixation -and the truncation of *nif*U, all proteins for nitrogen fixation are conserved in the region without signs of degenerative genome evolution. This conservation of *nif *genes is consistent with the hypothesis that molecular nitrogen fixation has been an important driving force for the endosymbiotic interaction.

It can be expected that endosymbiont and host biochemistry will change with the development of the symbiotic interaction. Genes whose products become superfluous for symbiont-host coexistence are expected targets for mutation. At earlier stages of accumulation of deleterious mutation, holomologues will still be identifiable by BLAST homology searches. Table 2 lists many pseudogenes that may fit this category.

## Conclusion

A diverse range of genetic modifications have occurred in the genome of *R. gibba *spheroid bodies and these would compromise the ability of the endosymbiont to exist as a free-living cyanobacterium, thereby confirming their suspected obligate status. Our findings provide insight into the genome evolution of a nitrogen-fixing endosymbiontic cyanobacterium living within a unicellular eukaryotic host. These are of special importance, as past inferences about processes of reductive genome evolution have mainly been based on the study of insect-bacteria interactions. In these, the symbionts reside within special cells or organs and thus their genomes may haven been subject to selection pressures different from those acting on the genomes of intracellular endosymbionts found in unicellular host organisms. Further analysis of the whole spheroid body's genome and comparison with free-living cyanobacteria will provide additional important information on the age of the interaction and the importance of different molecular processes and genetic modifications. Since the spheroid body is derived from cyanobacterial-like ancestors, the interaction could also serve as useful model system for understanding early events in the evolution of chloroplast genomes.

## Methods

### Symbiont Isolation and Purification

Spheroid bodies of *R. gibba *were isolated and DNA was purified as described by Prechtl [18]. *Cyanothece *sp. ATCC 51142 genomic DNA was isolated using standard procedures [33].

### Construction of gDNA libraries

Fosmid libraries of spheroid bodies and *Cyanothece *sp. ATCC 51142 were prepared using the *fosmid library construction kit *(Epicentre). After physical shearing, the DNA was blunt-end repaired and gel-fractionated to a fragment size of approximately 40 kbp as described by the manufacturer. Insert-DNA was ligated in the pCC1-Fos™ vector, constructs were in vitro packaged into phage particles and transfected into *Escherichia coli *EPI 300™-T1^R^.

### Screening for *nif*-gene region, *rec*A and *rec*F

Screening for clones containing the nitrogen fixing operon and flanking sequences and clones containing the *rec*A and *rec*F genes was performed using colony-PCR screening with oligonucleotides specific for spheroid body and the *Cyanothece nif*D-, *rec*A- and *rec*F-gene, respectively. Primer sequences were SBnifD_uni: 5'-CGG ACA AAG AAA ACG CAG AAT TTG-3', SBnifD_rev: 5'-CAG AAC GTC ATC ACA CTG TTT TTG-3', CynifD_uni: 5'-CCG TCA CGT TGT TCC TGC TTT C-3' CynifD_rev: 5'-CCA AGG GGT GCC AAT TAA TCC C-3', SBrecA_uni: 5'-CTA CTC TCG CTC TCC ATG CGA TTG-3', SBrecA_rev: 5'-CGG CGA ATA TCT AAA CGG ACT GAG-3', CyrecA_uni: 5'-GAT CGC AGA GGT GCA AAA GGC TG-3', CyrecA_rev: 5'-CAG TTC CTC CGG TGG TGA CTT C-3', SBrecF_uni: 5'-TCG GAC CTC AGC ATT ATC-3', SBrecF_rev: 5'-TCG ATG AGGTCC TAC TAA GC-3', CyrecF_uni: 5'-GCC GTC GAA TTA TTA GCA ACC C-3' and CyrecF_rev: 5'-GAA TTC GAC ATC ATC TCG ATG GG-3'. Clones for the upstream and downstream regions of positive *nif*-fosmids were obtained using the primers F13A12/3_uni: 5'-GAA CTC TAC AAT ACA GAT TAA CCG C-3', F13A12/3_rev: 5'-CAC TAA TCC ATC TAG ATT AGC CAC T-3', F13A12/5_uni: 5'-GGG CAT TCC AGA ATT AGA AGT AGG-3' and F13A12/5_rev: 5'-CTG TAG CCA AGC CAA AGT CGT TAT G-3' for the spheroid bodies and F4D10/5_uni: 5'-CAA GCT GTC TTT GGA CAA AAG-3', F4D10/5_rev: 5'-CGT TGA AGG TTT CCT CAA AAC-3', F4D10/3_uni: 5'-GAT ATC GTT GAA ACC TAT CGA G-3' and F4D10/3_rev: 5'-GAA TGT TAG GAC GAG CAA AAG G-3'. PCR reactions were performed using standard procedures.

### Cloning of positive fosmids

Preparation of fosmids and other DNA was performed according to standard protocols [33]. For subcloning, fosmid DNA was physically sheared by sonification. After blunt-end repair using the DNA Terminator Kit (Lucigen) and size fractionation by gel electrophoresis, fragments between 1000 and 1500 bp were isolated. The fragments were ligated in the pEZSeq™-vector (Lucigen) as described by the manufacturer and used to transform *E. coli *XL1blue MRF' cells. Sequencing of shotgun plasmids was carried out using cycle sequencing with 700 and 800 nm fluorescent labeled oligonucleotides and the LICOR™ sequencing system. 5'- and 3'-end sequencing of positive fosmid clones was performed using the primers M13 (700): 5'-GTA AAA CGA CGG CCA GT-3' and a modified pCC1™/pEpiFOS™ RP-2 (800): 5'-GCC AAG CTA TTT AGG TGA G-3'. Shotgun inserts in the pEZSeq™-vector were sequenced with M13_for: 5'-AGC GGA TAA CAA TTT CAC ACA GGA-3' and M13_rev: 5'-CGC CAG GGT TTT CCC AGT CAC GAC-3'.

### PCR analysis of missing or pseudogenised genes in the spheroid body's genome

PCR analysis of missing or pseudogenised genes in the spheroid body's genome was performed with primers specific for cyanobacterial *fdx*N, *pet*J, *psb*C, *cyl*0012 and *cyl*0017 genes. Primer sequences were fdxNuni: 5'-AGT TAC ACT ATC ACC AAT G-3', fdxNrev: 5'-ATT TCT TGG GAG TAA GCA TC-3', CYpetJuni: 5'-ATG AAA AGA TTA TTG TCC CT-3', CYpetJrev: 5'-TGC TTG ACT TAA RAC ATA AG-3', psbCuni: 5'-ACG TAG TTA AAG GAG TTA ACG-3', psbCrev: 5'-TTC GGC TAT CTG CTG AAA GC-3', CY0012uni: 5'-CCT CTC AAC TTA GCC ATT AG-3', CY0012rev: 5'-AAG CTT TGC TGT GTA GAA AC-3', CY0017uni: 5'-ATN RTN GGN TGY MGN AAY AA-3' and CY0017rev: 5'-GCD ATN ARN SHR TCN GGD AT-3'. CYrecF and SBrecF were used as positive controls, with the same primers used for the fosmid screening experiments. PCR reactions were performed using standard procedures.

### Sequence homology determinations and annotation

We assembled, finished and annotated sequences using the Sequencher [34] and Sequin software to allocate data and facilitate annotation. Identification of *orfs *was accomplished using BLAST analyses. Comparison of genome fragments was performed using BLAST analysis and the GATA tool for comparative sequence analysis [35]. Pseudogenes with one or more mutations were identified by BLAST searches or direct analysis of all open reading frames. Genome fragments of both *Cyanothece *sp. ATCC 51142 and *R. gibba *spheroid bodies are annotated in GenBANK under the accession numbers AY728386 and AY728387, respectively. Genes and *orfs *identified in both organisms were named according to BLASTp protein homologue names. Those *orfs *with homology to uncharacterised conserved hypothetical proteins and hypothetical *orfs *without any BLAST hit are numbered and referred as conserved hypothetical proteins and hypothetical proteins, respectively. *Orfs *oriented in the forward or reverse direction of the analysed fragment are named *cyl *or *cyr *for *Cyanothece*, *sbl *or *sbr *for spheroid bodies and follow consecutive numbering (see additional files 1 and 2).

### Phylogenetic tree building

Orthologues for spheroid body proteins greater than 200 amino acids from the genome region shown in Figure 1 were identified in closely related cyanobacteria using BLAST. These were aligned using CLUSTALX [34] and edited so that only conserved blocks of residues were used for evolutionary tree building. PHYML [36] was used to build maximum likelihood trees, assuming a JTT model of substitution and non-parametric bootstrapping (100 replicates). Strict consensus trees were built for each gene using the 100 gene trees produced from bootstrap replicates. SplitsTree 4.0 [37] was then used to build the supernetworks shown in Figure 2. Some proteins greater than 200 amino acids in length did not produce resolved strict consensus trees or were problematic for other reasons and these were omitted from the analysis (these included DapF, PyrF, Sbr0016, Sbl0019, FeoB1, NifP and Sbl0010). Protein sequences, additional to those reported in the present study and used for phylogenetic anlayses were those inferred from nucleotide sequences in both complete and unfinished genome projects. Genbank accession numbers for these are: *Cyanothece *sp. CCY0110 (AAXW00000000), *Crocosphaera watsonii *WH 8501 (AADV00000000), *Nodularia spumigena *CCY9414 (NZ_AAVW00000000), *Nostoc punctiforme *PCC 73102 (NZ_AAAY00000000), *Nostoc *sp. PCC 7120 (NC_003272), *Anabaena variabilis *ATCC 29413 (NC_007413), *Lyngbya *sp. PCC 8106 (NZ_AAVU00000000), *Trichodesmium erythraeum *IMS101 (NC_008312), *Synechocystis *sp. PCC 6803 (NC_000911).

## Authors' contributions

CK and CV performed the molecular studies, sequences alignments and drafted the manuscript. PL participated in drafting the manuscript and performed the phylogenetic analyses. UGM conceived of the study, participated in its design and coordination and helped to draft the manuscript. All authors read and approved the final manuscript.

## Supplementary Material

Additional File 1 BlastP analysis of identified and annotated *orf*s of *Cyanothece *sp. ATCC 51142 (accession number AY728386). The table provides information on all annotated *orfs *of the analysed *Cyanothece *sp. ATCC 51142 genome fragment. Click here for file

Additional File 2 BlastP analysis of identified and annotated *orf*s of *Rhopalodia gibba *spheroid bodies (accession number AY728387). The table provides information on all annotated *orfs *of the analysed spheroid body fragment genome fragment. Click here for file
