# Supplementary material for: The cyanobacterial endosymbiont of the unicellular algae Rhopalodia gibba shows reductive genome evolution
Source: BMC Evol Biol. 2008 Jan 28;8:30. doi: 10.1186/1471-2148-8-30 (PMC2246100; doi:10.1186/1471-2148-8-30)
Supplement: Additional File 1 — BlastP analysis of identified and annotated orfs of Cyanothece sp. ATCC 51142 (accession number AY728386). The table provides information on all annotated orfs of the analysed Cyanothece sp. ATCC 51142 genome fragment. [file 1471-2148-8-30-S1.doc]

| **No.** | **Name** | **Best BlastP Hit (complete bacterial genomes)** | **Species** | **Accession** | **e-value** |
| --- | --- | --- | --- | --- | --- |
| cy1  cy2  cy3  cy4  cy5  cy6  cy7  cy8  cy9  cy10  cy11  cy12  cy13  cy14  cy15  cy16  cy17  cy18  cy19  cy20  cy21  cy22  cy23  cy24  cy25  cy26  cy27  cy28  cy29  cy30  cy31  cy32  cy33  cy34  cy35  cy36  cy37  cy38  cy39  cy40  cy41  cy42  cy43  cy44  cy45  cy46  cy47  cy48  cy49  cy50  cy51  cy52  cy53  cy54  cy55  cy56  cy57  cy58  cy59  cy60  cy61  cy63  cy64  cy65  cy66  cy67  cy68  cy69 | *cyl*0001/*cor*A  *cyr*0002  *cyr*0003  *nif*T  *nif*Z  *nif*V  *cyr*0004  *cyr*0005  *nif*P  *nif*B  fdxN  *nif*S  *nif*U  *nif*H  *nif*D  *nif*K  *cyl*0006  *nif*E  *nif*N  *nif*X  *orf*2  *orf*1  *nif*W  *hes*A  *hes*B  *fdx*H  *cyl*0007  *cyl*0008  *cyl*0009/feoB1  *cyl*0010/feoB2  *cyl*0011/feoA  *cyl*0012  *cyl*0013/modC  *fdx*III  *cyl*0014/vagC  *cyl*0015/pilT  *cyl*0016  *cyl*0017/vapC  *cyl*0018  *cyl*0019  *cyl*0020  *cyl*0021  *cyl*0022/tpr  petJ  petE  aldolase  *cyr*0024  peptidase  *pro*B  *glk*  *mrr*  *cyl*0027  *cyr*0028/slh  *cyr*0029  *gpp*A  *rpo*D  *cyr*0030  *ndh*F  *ndh*D  *cyl*0031  *pgm*  *cyl*0033  *cyl*0034/tonB  *cyr*0035  *cob*N  *cyr*0036  *cyl*0038  *cyl*0039 | Magnesium and cobalt transport protein CorA  Nitrogenase-associated protein  hypothetical protein CY0110_22347  NifT/FixU  NifZ  trans-homoaconitate synthase  hypothetical protein CY0110_22367  hypothetical protein CY0110_22372  serine acetyltransferase  Nitrogenase cofactor biosynthesis protein  4Fe-4S ferredoxin, iron-sulfur binding protein  nitrogenase cofactor synthesis protein  Fe-S cluster assembly protein NifU  Nitrogenase iron protein  Nitrogenase molybdenum-iron protein alpha chain  Nitrogenase molybdenum-iron protein beta chain  Mo-dependent nitrogenase-like protein  Nitrogenase MoFe cofactor biosynthesis protein  Nitrogenase MoFe cofactor biosynthesis protein  Dinitrogenase iron-molybdenum cofactor biosynthesis  hypothetical protein CY0110_22447  hypothetical protein CY0110_22452  Nitrogen fixation protein NifW  MoeZ/MoeB  HesB protein  Ferredoxin (2Fe-2S)  hypothetical protein CY0110_22477  hypothetical protein CY0110_22482  iron(II) transporter  iron(II) transporter  FeoA family protein  hypothetical protein CY0110_22502  Molybdate ABC transporter  4Fe-4S ferredoxin  hypothetical protein CwatDRAFT_6492  PilT protein, N-terminal  hypothetical protein CwatDRAFT_6492  PIN domain protein  conserved hypothetical protein  hypothetical protein L8106_01787  hypothetical protein CY0110_28344  hypothetical protein CY0110_28339  TPR repeat protein  Cytochrome c, class I  plastocyanin precursor  keto-hydroxyglutarate-aldolase  keto-hydroxyglutarate-aldolase  S8putative protease  gamma-glutamyl kinase  glucokinase  mrr restriction system protein  hypothetical protein CY0110_28284  hypothetical protein CY0110_28279  hypothetical protein Ava_C0220  exopolyphosphatase  RNA polymerase sigma factor  hypothetical protein CY0110_29084  NAD(P)H-quinone oxidoreductase subunit F  NAD(P)H-quinone oxidoreductase subunit D3  hypothetical protein CY0110_29059  phosphoglucomutase  hypothetical protein CY0110_24621  hypothetical protein CY0110_24616  hypothetical protein CY0110_07524  Magnesium-chelatase, subunit H  hypothetical protein RS9916_38322  hypothetical protein CY0110_22392  hypothetical protein CY0110_07519 | *Cyanothece* sp. CCY0110  *Cyanothece* sp. CCY0110  *Cyanothece* sp. CCY0110  *Cyanothece* sp. CCY0110  *Cyanothece* sp. CCY0110  *Cyanothece* sp. CCY0110  *Cyanothece* sp. CCY0110  *Cyanothece* sp. CCY0110  *Cyanothece* sp. CCY0110  *Cyanothece* sp. CCY0110  *Cyanothece* sp. CCY0110  *Cyanothece* sp. CCY0110  *Cyanothece* sp. CCY0110  *Cyanothece* sp. CCY0110  *Cyanothece* sp. CCY0110  *Cyanothece* sp. CCY0110  *Cyanothece* sp. CCY0110  *Cyanothece* sp. CCY0110  *Cyanothece* sp. CCY0110  *Cyanothece* sp. CCY0110  *Cyanothece* sp. CCY0110  *Cyanothece* sp. CCY0110  *Cyanothece* sp. CCY0110  *Cyanothece* sp. CCY0110  *Cyanothece* sp. CCY0110  *Cyanothece* sp. CCY0110  *Cyanothece* sp. CCY0110  *Cyanothece* sp. CCY0110  *Cyanothece* sp. CCY0110  *Cyanothece* sp. CCY0110  *Cyanothece* sp. CCY0110  *Cyanothece* sp. CCY0110  *Cyanothece* sp. CCY0110  *Cyanothece* sp. CCY0110  *Crocosphaera watsonii* WH8501  *Crocosphaera watsonii* WH8501  *Crocosphaera watsonii* WH8501  *Lyngbya* sp. PCC 8106  *Cyanothece* sp. CCY0110  *Lyngbya* sp. PCC 8106  *Cyanothece* sp. CCY0110  *Cyanothece* sp. CCY0110  *Cyanothece* sp. CCY0110  *Cyanothece* sp. CCY0110  *Cyanothece* sp. CCY0110  *Cyanothece* sp. CCY0110  *Cyanothece* sp. CCY0110  *Cyanothece* sp. CCY0110  *Cyanothece* sp. CCY0110  *Cyanothece* sp. CCY0110  *Cyanothece* sp. CCY0110  *Cyanothece* sp. CCY0110  *Cyanothece* sp. CCY0110  *Anabaena variabilis* ATCC 29413  *Cyanothece* sp. CCY0110  *Cyanothece* sp. CCY0110  *Cyanothece* sp. CCY0110  *Cyanothece* sp. CCY0110  *Cyanothece* sp. CCY0110  *Cyanothece* sp. CCY0110  *Cyanothece* sp. CCY0110  *Cyanothece* sp. CCY0110  *Cyanothece* sp. CCY0110  *Cyanothece* sp. CCY0110  *Cyanothece* sp. CCY0110  Synechococcus sp. RS9916  *Cyanothece* sp. CCY0110  *Cyanothece* sp. CCY0110 | ZP_01727449.1  ZP_01727751.1  ZP_01727752.1  ZP_01727753.1  ZP_01727754.1  ZP_01727755.1  ZP_01727756.1  ZP_01727757.1  ZP_01727758.1  ZP_01727760.1  ZP_01727762.1  ZP_01727763.1  ZP_01727764.1  ZP_01727765.1  ZP_01727766.1  ZP_01727767.1  ZP_01727768.1  ZP_01727769.1  ZP_01727770.1  ZP_01727771.1  ZP_01727772.1  ZP_01727773.1  ZP_01727774.1  ZP_01727775.1  ZP_01727776.1  ZP_01727777.1  ZP_01727778.1  ZP_01727779.1  ZP_01727780.1  ZP_01727781.1  ZP_01727782.1  ZP_01727783.1  ZP_01727784.1  ZP_01727785.1  ZP_00514029.1  ZP_00514028.1  ZP_00514029.1  ZP_01621173.1  ZP_00514027.1  ZP_01619026.1  ZP_01728264.1  ZP_01728263.1  ZP_01728262.1  ZP_01728261.1  ZP_01728260.1  ZP_01728259.1  ZP_01728258.1  ZP_01728257.1  ZP_01728256.1  ZP_01728255.1  ZP_01728254.1  ZP_01728252.1 ZP_01728251.1  YP_320494.1  ZP_01728249.1  ZP_01730799.1  ZP_01730798.1  ZP_01730795.1  ZP_01730794.1  ZP_01730793.1  ZP_01728338.1  ZP_01728337.1  ZP_01728336.1  ZP_01728777.1  ZP_01728199.1  ZP_01471695.1  ZP_01727761.1  ZP_01728776.1 | 5e-106  1e-73  2e-103  2e-30  3e-35  0.0  4e-34  6e-50  4e-132  0.0  3e-66  0.0  8e-128  3e-148  0.0  0.0  7e-46  0.0  0.0  1e-73  2e-76  1e-34  6e-56  1e-127  4e-60  3e-50  1e-73  1e-52  2e-107  0.0  6e-37  1e-76  0.0  3e-40  2e-14  2e-23  2e-21  2e-64  3e-24  1e-39  6e-99  7e-121  2e-32  4e-53  2e-103  9e-28  0.0  0.0  1e-167  8e-85  0.0  0.0  4e-49  0.0  4e-175  5e-32  0.0  0.0  0.0  0.0  1e-33  5e-153  2e-101  0.0  3e-15  5e-22  5e-16 |

**Additional file 1: BlastP analysis of identified and annotated *orf*s of *Cyanothece* sp. ATCC 51142 (accession number AY728386).**
