# Supplementary material for: The cyanobacterial endosymbiont of the unicellular algae Rhopalodia gibba shows reductive genome evolution
Source: BMC Evol Biol. 2008 Jan 28;8:30. doi: 10.1186/1471-2148-8-30 (PMC2246100; doi:10.1186/1471-2148-8-30)
Supplement: Additional File 2 — BlastP analysis of identified and annotated orfs of Rhopalodia gibba spheroid bodies (accession number AY728387). The table provides information on all annotated orfs of the analysed spheroid body fragment genome fragment. [file 1471-2148-8-30-S2.doc]

**Additional file 2: BlastP analysis of identified and annotated *orf*s of *Rhopalodia gibba* spheroid bodies (accession number AY728387).**

| **No.** | **Name** | **Best BlastP Hit (complete bacterial genomes)** | **Organism** | **Accession-No.** | **e-value** |
| --- | --- | --- | --- | --- | --- |
| sb1  sb2  sb3  sb4  sb5  sb6  sb7  sb8  sb9  sb10  sb11  sb12  sb13  sb14  sb15  sb16  sb17  sb18  sb19  sb20  sb21  sb22  sb23  sb24  sb25  sb26  sb27  sb28  sb29  sb30  sb31  sb32  sb33  sb34  sb35  sb36  sb37  sb38  sb39  sb40  sb41  sb42  sb43  sb44  sb45  sb46  sb47  sb48 | *nif*Z  *nif*V  *sbr*0001  *sbr*0002  *nif*P  *nif*B  *nif*S  *nif*U  *nif*H  *nif*D  *nif*K  *nif*E  *nif*N  *nif*X  *orf*2  *orf*1  *nif*W  *hes*A  *hes*B  *fdx*H  *sbl*0003  *sbl*0004  *sbl*0005  *sbl*0006/feoB1  *sbl*0007/feoB2  *sbl*0008/feoA  *sbl*0009/*mod*C  *fdx*III  *sbl*0010  *sbr*0011/*SAM*  *sbl*0012  *sbr*0013  *ndh*J  *ndh*K  *ndh*C  *sbr*0014  *sbr*0015/*ABC*  *sbr*0016  *sbr*0017  *dap*F  *sbl*0019  *sbl*0020  *sbl*0021  *sbr*0022/*rfa*G  *pyr*F  *sbl*0023  *sbl*0024/*per*M  *sbr*0025/*uvr*D | NifZ  trans-homoaconitate synthase  hypothetical protein CwatDRAFT_3844  hypothetical protein CY0110_22372  serine acetyltransferase  Nitrogenase cofactor biosynthesis protein  Aromatic amino acid beta-eliminating lyase  Fe-S cluster assembly protein NifU  Nitrogenase iron protein  Nitrogenase molybdenum-iron protein alpha  Nitrogenase molybdenum-iron protein beta chain  Nitrogenase MoFe cofactor biosynthesis protein  Nitrogenase MoFe cofactor biosynthesis protein  Nitrogen fixation-related protein  hypothetical protein CY0110_22447  Protein of unknown function DUF683  Nitrogen fixation protein NifW  MoeZ/Mo  HesB protein  Ferredoxin (2Fe-2S)  hypothetical protein CY0110_22477  hypothetical protein CY0110_22482  hypothetical protein L8106_11022  iron(II) transporter  iron(II) transporter  FeoA family protein  Molybdate ABC transporter, permease protein  4Fe-4S ferredoxin, iron-sulfur binding protein  conserved hypothetical protein  hypothetical protein CY0110_15517  conserved hypothetical protein  Protein of unknown function DUF1499  NADH dehydrogenase (ubiquinone), 30 kDa subunit  NADH dehydrogenase subunit B  NADH-ubiquinone/plastoquinone oxidoreductase  hypothetical protein CY0110_01964  ABC transporter  hypothetical protein CY0110_11207  Penicillinase repressor  Diaminopimelate epimerase  two-component response regulator  hypothetical protein CY0110_21717  hypothetical protein CY0110_12192  hypothetical protein CY0110_04628  Orotidine 5'-phosphate decarboxylase  hypothetical protein CY0110_10137  hypothetical protein CY0110_29024  UvrD/REP helicase | *Crocosphaera watsonii* WH 8501  *Cyanothece* sp. CCY0110  *Crocosphaera watsonii* WH 8501  *Cyanothece* sp. CCY0110  *Cyanothece* sp. CCY0110  *Crocosphaera watsonii* WH 8501  Lyngbya sp. PCC 8106  *Cyanothece* sp. CCY0110  *Cyanothece* sp. CCY0110  *Crocosphaera watsonii* WH 8501  *Cyanothece* sp. CCY0110  *Cyanothece* sp. CCY0110  *Cyanothece* sp. CCY0110  *Crocosphaera watsonii* WH 8501  *Cyanothece* sp. CCY0110  *Crocosphaera watsonii* WH 8501  *Crocosphaera watsonii* WH 8501  *Crocosphaera watsonii* WH 8501  *Cyanothece* sp. CCY0110  Lyngbya sp. PCC 8106  *Cyanothece* sp. CCY0110  *Cyanothece* sp. CCY0110  *Lyngbya* sp. PCC 8106  *Cyanothece* sp. CCY0110  *Cyanothece* sp. CCY0110  *Cyanothece* sp. CCY0110  *Cyanothece* sp. CCY0110  *Cyanothece* sp. CCY0110  *Crocosphaera watsonii* WH 8501  *Cyanothece* sp. CCY0110  *Crocosphaera watsonii* WH 8501  *Crocosphaera watsonii* WH 8501  *Crocosphaera watsonii* WH 8501  *Cyanothece* sp. CCY0110  *Crocosphaera watsonii* WH 8501  *Cyanothece* sp. CCY0110  *Crocosphaera watsonii* WH 8501  *Cyanothece* sp. CCY0110  *Crocosphaera watsonii* WH 8501  *Crocosphaera watsonii* WH 8501  *Cyanothece* sp. CCY0110  *Cyanothece* sp. CCY0110  *Cyanothece* sp. CCY0110  *Cyanothece* sp. CCY0110  *Crocosphaera watsonii* WH 8501  *Cyanothece* sp. CCY0110  *Cyanothece* sp. CCY0110  *Crocosphaera watsonii* WH 8501 | ZP_00516413.1  ZP_01727755.1  ZP_00516411.1  ZP_01727757.1  ZP_01727758.1  ZP_00516383.1  ZP_01620771.1  ZP_01727764.1  ZP_01727765.1  ZP_00516387.1  ZP_01727767.1  ZP_01727769.1  ZP_01727770.1  ZP_00516392.1  ZP_01727772.1  ZP_00516394.1  ZP_00516395.1  ZP_00516396.1  ZP_01727776.1  ZP_01620755.1  ZP_01727778.1  ZP_01727779.1  ZP_01620752.1  ZP_01727780.1  ZP_01727781.1  ZP_01727782.1  ZP_01727784.1  ZP_01727785.1  ZP_00514027.1  ZP_01726472.1  ZP_00515293.1  ZP_00514942.1  ZP_00518733.1  ZP_01728727.1  ZP_00518731.1  ZP_01728083.1  ZP_00516248.1  ZP_01730911.1  ZP_00514471.1  ZP_00514083.1  ZP_01729947.1  ZP_01727626.1  ZP_01731170.1  ZP_01730205.1  ZP_00516222.1  ZP_01726321.1  ZP_01732518.1  ZP_00517150.1 | 5e-40  5e-165  4e-23  5e-21  1e-93  0.0  1e-169  2e-23  4e-143  0.0  0.0  0.0  0.0  1e-49  2e-63  8e-21  3e-38  2e-81  7e-42  6e-38  4e-52  1e-29  0.56  6e-73  2e-162  1e-27  0.0  2e-33  7e-57  0.0  2e-34  3e-41  8e-88  5e-127  1e-50  3e-143  2e-123  1e-74  3e-55  4e-104  2e-85  2e-26  2e-61  3e-159  5e-82  1e-50  2e-116  0.0 |
